# The function and mechanisms of action of circular RNAs in Urologic Cancer

**DOI:** 10.1186/s12943-023-01766-2

**Published:** 2023-03-25

**Authors:** Zi-hao Zhang, Yue Wang, Ya Zhang, Sheng-Feng Zheng, Tao Feng, Xi Tian, Mierxiati Abudurexiti, Zhen-Da Wang, Wen-Kai Zhu, Jia-Qi Su, Hai-Liang Zhang, Guo-Hai Shi, Zi-Liang Wang, Da-Long Cao, Ding-Wei Ye

**Affiliations:** 1 Qingdao Institute, School of Life Medicine, Department of Urology, Fudan University Shanghai Cancer Center, Fudan University, Qingdao, 266500 China; 2 Department of Urology, State Key Laboratory of Genetic Engineering, Collaborative Innovation Center for Genetics and Development, School of Life Sciences, Fudan University Shanghai Cancer Center, Fudan University, Shanghai, 200433 China; 3 Department of Urology, Fudan University Shanghai Cancer Center, No. 270 Dong’an Road, Shanghai, 200032 People’s Republic of China; 4 Department of Nephrology, Xin Hua Hospital, Shanghai Jiao Tong University School of Medicine, 1665 Kongjiang Road, Shanghai, 200092 China; 5 Shanghai Pudong New Area Gongli Hospital, Shanghai, 200135 China; 6 Institute of Cancer Research, Department of Gynecology, Shanghai Municipal Hospital of Traditional Chinese Medicine, Shanghai University of Traditional Chinese Medicine, Shanghai, 200071 P. R. China

**Keywords:** CircRNA, Kidney cancer, Bladder cancer, And prostate cancer

## Abstract

Kidney, bladder, and prostate cancer are the three major tumor types of the urologic system that seriously threaten human health. Circular RNAs (CircRNAs), special non-coding RNAs with a stabile structure and a unique back-splicing loop-forming ability, have received recent scientific attention. CircRNAs are widely distributed within the body, with important biologic functions such as sponges for microRNAs, as RNA binding proteins, and as templates for regulation of transcription and protein translation. The abnormal expression of circRNAs in vivo is significantly associated with the development of urologic tumors. CircRNAs have now emerged as potential biomarkers for the diagnosis and prognosis of urologic tumors, as well as targets for the development of new therapies. Although we have gained a better understanding of circRNA, there are still many questions to be answered. In this review, we summarize the properties of circRNAs and detail their function, focusing on the effects of circRNA on proliferation, metastasis, apoptosis, metabolism, and drug resistance in kidney, bladder, and prostate cancers.

**Supplementary Information:**

The online version contains supplementary material available at 10.1186/s12943-023-01766-2.

## Introduction

Prostate, renal, and bladder cancer (BCa) are the most common tumors of the urinary system [1]. Urinary system tumors account for approximately 10% of total tumor incidence, with prostate cancer accounting for 29% of male cancer cases [2]. Surgery is currently the most effective treatment for urological tumors, but the 5-year survival rate is still unsatisfactory [3, 4]. For locally progressive and metastatic prostate cancer (PCa), androgen ablation is usually the best treatment option [5]. This treatment option effectively extends overall patient survival [6]. Targeted and immune checkpoint therapies have been shown to improve the prognosis for renal cell carcinoma (RCC) patients, with tyrosine kinase inhibitors widely used for clinical treatment [7, 8]. However, patients with BCa have a high recurrence rate and are prone to distant metastases. Patients with advanced or metastatic BCa have a poor prognosis [9]. Post-operative supplementation with bladder instillation of BCG or gemcitabine significantly improves the quality of life for patients with BCa and effectively reduce the recurrence rate [10, 11]. Although there have been many improvements in BCa treatment, early diagnosis of disease results in the best possible patient outcomes. Therefore, identification of novel biomarkers for tumor is a significant and current clinical objective, with circRNAs promising biomarkers for disease [12, 13].

Non-coding RNAs (ncRNAs) are a class of RNA that are not translated into proteins and are usually classified by size into small RNAs such as microRNA (miRNA) (about 22nt) and long-stranded non-coding RNAs (lncRNA, > 200 nt) [14]. NcRNAs are involved in tumor development and play an important role in disease [15]. CircRNAs are a family of naturally occurring long non-coding RNAs that are widely expressed in eukaryotes. Covalently closed circular RNA molecules were first discovered in 1976 within viroids [16]. Subsequently, circRNAs were found to exist in the cytoplasm of eukaryotic cells [3] and to be expressed in a cell- and organ-specific manner with important biological functions [17, 18]. With the development and popularization of sequencing technology, the importance of circRNAs has become clearer. As a special lncRNA, circRNAs have a unique covalent single-stranded closed-loop structure, which lack a 5’ cap or 3’ poly (A) tail [19]. Compared to mRNAs, circRNA have a more stable structure and are not easily degraded by RNase A. Recent studies have shown that circRNAs play important roles in many cancers by; acting as microRNA sponges [20], interacting with RNA-binding proteins [21], gene transcriptional regulation, alternative splicing [22–24], and protein translation [25].

In this review, we briefly describe the principles of circRNA formation and summarize the impact of circRNAs on the occurrence and development of RCC, PCa, and BCa. Further, the use of circRNAs as diagnostic and prognostic biomarkers is discussed. A deeper understanding of circRNAs will provide valuable clues and useful information for future urologic cancer treatment.

## Overview of CircRNAs

In 1976, Sanger initiated the study of circRNAs [16]. Nigro et al. were the first to identify circular RNA transcripts in mammals - a phenomenon of “scrambled exons” in which exons were linked end-to-end to form a circular structure [26]. With improved technology and in-depth research, ncRNAs, once considered a “miscellaneous signal”, have been extensively studied. CircRNAs have a unique circular structure. Compared with linear RNAs, circRNAs are more stable and resistant to degradation by nucleic acid exonucleases [27]. The structure is highly conserved throughout development and evolution.

### Biogenesis and regulation of CircRNAs

**Fig. 1:**
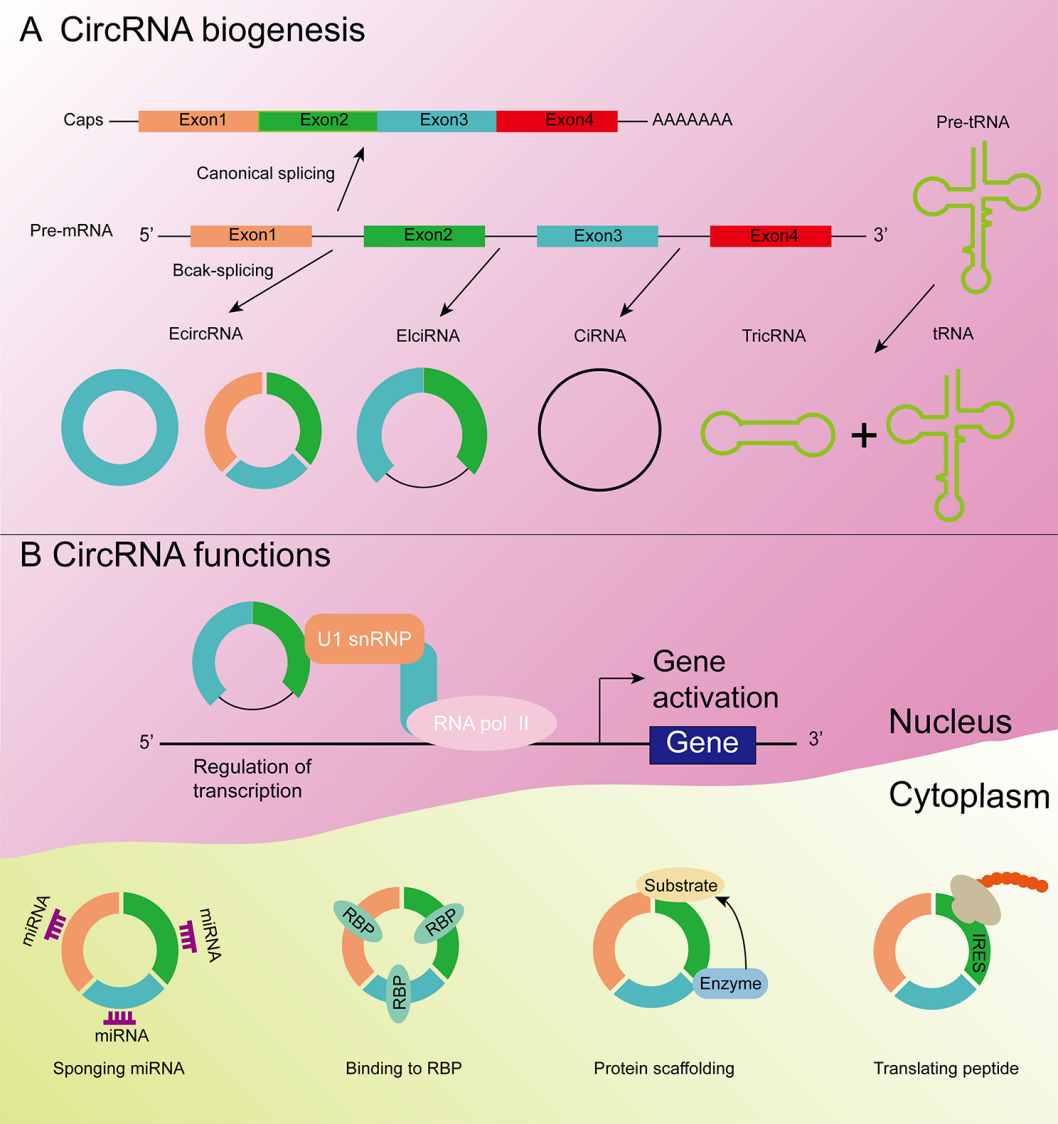
Biogenesis and function of circular RNA (circRNA) Pre-mRNA can undergo either canonical splicing to generate a linear RNA with exon inclusion or back-splicing to produce both a circular RNA and an alternatively spliced linear RNA with exon exclusion. (A) The biogenesis of circRNAs. Biogenesis of circRNAs generates three types of circRNAs: EcircRNAs, ciRNAs, and EIciRNAs. The formation of ecircRNAs is usually by direct back splicing. Direct back splicing is a common shearing method in which precursor-mRNA is looped by linking the 5′ splice donor site downstream of the exon to the 3′ splice acceptor site upstream via covalent bonding. EIcircRNAs also use the method of direct back splicing, but in the process of back splicing, some intron sequences are not removed but remain within the circRNA. The formation of ciRNAs is thought to result from lasso formation from introns removed during pre-mRNA splicing. TricRNA is a circular RNA produced by tRNA precursor splicing. (B) The function of circRNAs. CircRNAs enhance the transcription and splicing of their parental genes by interacting with RNA pol II or U1 small nuclear ribonucleoproteins (U1 snRNPs). CircRNAs have basic functions as microRNA (miRNA) sponges, RNA-binding proteins (RBP), and templates for regulating transcription and protein translation. CircRNAs can function as microRNA sponges or decoys, protecting target mRNAs from miRNA-dependent degradation. circRNAs containing RBP binding motifs may function as sponges or decoys for these proteins and indirectly regulate their functions. circRNAs have been shown to function as protein scaffolds, facilitating the colocalization of enzymes and their substrates. CircRNA with internal ribosomal entry site elements may be translated under certain circumstances to produce unique peptides

CircRNAs can be classified based on the method of splicing. Exon circRNAs (EcircRNAs), exon-intron circRNAs (EIcircRNAs), circular intron RNAs (ciRNAs), and tRNAintronic circular RNAs (tricRNAs) are formed after splicing of precursor transfer RNAs (pre-tRNAs) [28] (Fig. 1). EcircRNAs account for the majority of circRNAs and are mainly located within the cytoplasm [29]. EIcircRNAs and ciRNAs are primarily located within the nucleus [30]. The current common circRNA looping models are; direct back splicing, lasso-driven circularization, and RNA-binding protein-mediated circularization [31]. The formation of ecircRNAs is usually by direct back splicing. Direct back splicing is a common shearing method in which precursor-mRNA is looped by linking the 5′ splice donor site downstream of the exon to the 3′ splice acceptor site upstream via covalent bonding. The process forms a cord-tailed splice loop, which is followed by removal of the intron by shearing, producing a closed-loop circRNA [22]. EIcircRNAs also use the method of direct back splicing, but in the process of back splicing, some intron sequences are not removed but remain within the circRNA [32]. The formation of ciRNAs is thought to result from lasso formation from introns removed during pre-mRNA splicing. The formation of such ciRNAs can be recapitulated using expression vectors, wherein processing depends on a consensus motif containing a 7 nt GU-rich element near the 5′ splice site and an 11 nt C-rich element close to the branch point site [33].

RNA-binding proteins (RBPs) are also a means by which circRNAs are formed. RBPs include; muscle blind-like protein 1, the RNA-editing enzyme, quaking, and fusion sarcoma protein [34–37]. Exon loops can also be driven in a similar fashion by the dimerization of RBPs that bind to specific motifs within flanking introns [36]. In large-scale transcriptome analysis, 61% of human genes can generate both circular and linear transcripts [38, 39]. CircRNA biogenesis uses canonical splice sites, with back-splicing in competition with linear splicing of mRNAs. Transcript levels of circRNAs are generally at lower levels than mRNAs. Back splicing is reduced during normal physiological conditions, with decreased activity of spliceosomal components resulting in increased circRNA expression [40]. CircRNA exons and their flanking introns are much longer than linear RNAs [18]. These longer introns contain reverse complementary sequence elements (e.g. inverted-repeat Alu elements) that bring the downstream 5-donor and upstream 3-acceptor splice sites into proximity [41, 42].

### Detection and identification of CircRNAs

More than 100,000 different human circRNAs have been discovered in the past decades [43, 44]. However, the unique circular structure, low abundance, and lack of 5′-caps and 3′-poly (A) tails make it difficult to efficiently detect and identify circRNAs by commonly used techniques [45].

Northern blotting and PCR techniques have played an important role in the initial detection and discovery of circRNAs (Table 1) [46]. Northern blotting is the gold standard for circRNA validation by hybridization with RNA probes and separation of circRNAs into linear counterparts by denaturing polyacrylamide gel electrophoresis [33, 41, 47]. The disadvantages of northern blotting are a poor detection rate for low abundance circRNAs and the lack of a means for high-throughput detection [48]. RT-qPCR is the most commonly used method for circRNAs analysis using different primers that flank the BSJ locus for circRNA fragment amplification and circRNA detection [47, 49]. Due to strand displacement and rolling circle replication, circRNA molecules can produce false positive signals during reverse transcription [50].

Rolling circle amplification (RCA) is a recently developed technique wherein the circular structure of circRNAs is an ideal template for initiating the rolling circle amplification process, making this technique particularly suitable for circRNA analysis [51]. The reverse transcription RCA method selectively amplifies target circRNAs, with the advantages of ease of operation and low cost [52]. Enzyme-based circRNA detection reduces the need for reliance upon primers for detection of circRNAs. Duplex-specific nucleases (DSN) digest the DNA strand of a DNA/circRNA hybrid, releasing a fluorescent probe fragment and circRNA, which enhances the fluorescent signal in the presence of target circRNA [53]. NanoString Technologies nCounter assays are the latest method for accurate and sensitive detection of circRNA. NanoString uses dual probe hybridization with biotinylated capture probes and unique color-coded reporter probes to accurately quantify circRNA without enzymatic reactions or bias [54]. However, this technology requires specialized equipment that is expensive. Microarrays are also widely used as a high-throughput assay with good accuracy and sensitivity for circRNAs detection. These assays are not affected by low levels of transcript expression and are suitable for circRNA expression studies [55, 56]. However, the data generated by microarrays are difficult to compare between different studies [46]. Fluorescence in situ hybridization (FISH) is a common tool for studying the subcellular localization of RNA, using fluorescently labeled DNA probes [57, 58]. However, this technique is time-consuming and requires expensive signal detection equipment [46].

The development of RNA-seq technology has greatly advanced biomedical research as well as the detection of circRNAs [59, 60]. The low abundance of circRNAs in vivo and the lack of poly (A) tails require biochemical enrichment of circRNAs prior to short-read deep sequencing [45]. Treatment of samples with RNase R significantly enriches circRNAs [49, 61]. Performing rRNA depletion prior to sequencing and construction of circRNAs libraries, without polyA screening and transcriptome sequencing, is widely used for early circRNA analysis [62]. Ribo-RNA seq provides expression data for both coding and non-coding RNAs and has identified hundreds of circRNAs [24]. Ribo-RNA-seq has the advantage of direct quantitative comparison of the expression levels of circRNAs and their cognate linear RNAs [63]. However, this approach leads to overlapping signals of circRNAs and linear RNAs in exonic regions that cannot be separated [64]. Poly(A)-RNA-seq is suitable for circRNAs lacking poly(A) tails, and this technique has identified hundreds of human genes expressing circular RNA isoforms [65]. The limitations of this technique are that it may not detect small circRNAs and cannot accurately identify and quantify rare circular isoforms [65]. The RNase R RNA-seq method can identify more individual circRNAs than ribo- or poly (A)-RNA-seq, maximizing circRNA enrichment [41, 47]. There is a need for development of a simple, accurate, sensitive, and effective method for circRNA detection and analysis.

**Table 1:** Methods for detecting and quantifying circRNA

Method	Sensitivity /Accuracy	Throughput	Advantages and Disadvantages	Ref
Northern blotting	Low	Low	Gold standard analysis of circRNA validation;Insensitive to low expression of circRNA	[33, [41, 46–48]
RT-qPCR	Medium-High	Low-Medium	Provide quantitative data; False positive signal may be generated	[47, 49, 50]
RCA	High	Medium	Simple operation, no need for high-precision temperature cycle and additional separation steps.	[51, 52]
NanoString Technologies nCounter assays	High	Medium-High	Quantify circRNA;Requires special equipment and is expensive	[54]
Microarrays	Medium	High	High throughput and high detection efficiency;Difficult to compare between different studies	[46, 55, 56]
FISH	High	Low	Forming stable DNA-RNA hybrid;Highly affected by personnel operations and instrumentation and costly	[57, 58]
RNA-seq	Medium-High	High	Widely used in the discovery of novel circRNAs;Overlap with linear molecular signals, data processing capabilities are required	[59, 60, 62, 64]

### Functions of CircRNAs

#### CircRNAs act as MiRNA sponges

The most widely studied circRNA function is the capacity of circRNAs to serve as miRNA sponges. MiRNAs, an important class of small non-coding RNAs, can bind to the 3′ untranslated regions (3′ UTR) of mRNA, regulating gene expression, protein degradation, and translational repression [66]. By study of circRNA, circRNAs were found to bind miRNA and compete with mRNA for miRNA binding, serving as miRNA sponges and indirectly affecting gene expression [67, 68]. When circRNA adsorbs functional miRNA, it reduces the availability of miRNA and upregulates the expression of miRNA-targeted mRNA. The mRNA can then be recombined by the ribosome and converted into protein [24]. CiRS-7 is a canonical circRNA that acts as miRNA sponge that contains more than 70 miR-7 binding sites [69, 70]. A recent study found that ciRS-7 promotes the progression and metastasis of RCC through the PI3K/AKT signaling pathway by binding to miR-139-3p and blocking its inhibitory effect on TAGLN [71]. Many other circRNAs have also been shown to function as miRNA sponges. CircEYA3 exerts its oncogenic function by up-regulation of c-Myc expression through adsorption of miR-1294 [72]. Androgen receptor (AR) inhibits circHIAT1 expression, reducing expression of miR-195-5p/29a-3p/29c-3p, thereby increasing CDC42 expression, enhancing migration and invasion of Clear cell renal cell carcinomas (ccRCC) [73]. CircFAT1 contains miR-375 binding sites and adsorbs miR-375, up-regulating YES-associated protein 1 expression. Furthermore, inhibition of miR-375 reverses the attenuation of cell proliferation, migration, and invasion induced by circFAT1 knockdown, thereby promoting tumorigenesis [74].

#### CircRNAs affect splicing and regulate transcription

Alternative splicing in eukaryotes ensures the diversity of biological proteins. The biogenesis of circRNAs at the cellular level relies on typical splice sites and spliceosome mechanisms and competes with pre-mRNA splicing, which in turn affects the expression of transcribed genes [42]. Studies have found that circRNAs have the ability to transcribe and control transcription and gene expression during development and disease. EIcircRNAs and ciRNAs in the nucleus play a very important role in gene transcription. EICircRNAs interact with U1 small nuclear ribonucleoproteins (U1 snRNPs) and with the polymerase II complex, promoting transcription of their parent genes [75]. CircDONSON is expressed in the nucleus and positively related with a poor advanced TNM stage and poor prognosis for gastric cancer patients. CircDONSON recruits the NURF complex to initiate SOX4 expression, which promotes gastric cancer progression by direct interaction with the SNF2L subunit [76]. High expression of circRHOT1 in hepatocellular carcinoma (HCC) significantly promotes the growth and metastasis of HCC, with circRHOT1 recruiting TIP60 to the NR2F6 promoter, initiating NR2F6 transcription. Knockdown of circRHOT1 inhibits the transcription of NR2F6, and also inhibits the proliferation and metastasis of hepatoma cells [77].

#### CircRNAs function by interaction with proteins

In addition to serving as miRNA sponges and as a means by which to bind polymerase II complexes, circRNAs can also bind proteins. As a protein sponge, circRNA can regulate RNA indirectly, with some circRNAs containing only a few miRNA binding sites and are therefore particularly important for protein binding [42].

However, the RBP binding density of circRNA is less than that of linear mRNA [78]. CircFoxo3 is highly expressed in non-cancer cells and is associated with cell cycle progression. CircFoxo3 can bind CDK2 and P21 to inhibit cell cycle progression to form a ternary complex [79]. High expression of circAmotl1 in neonatal cardiac tissue can enhance AKT and enhance cardiomyocyte survival. CircAmotl1 binds to PDK1 and AKT1 to activate AKT phosphorylation and nuclear translocation [80].

#### CircRNAs undergo translation

Since circRNA acts as a non-coding RNAs, there is no free 5′ cap or 3′ poly(A) tail or easily recognizable open reading frame necessary for translation control [81]. Initially, circRNAs were thought to have no protein-coding ability, although some studies have shown circRNAs to code for protein. Due to the presence of a specific sequence, insertion of a synthetic internal ribosome entry site (IRES) upstream of the initial codon can directly recruit ribosome translation initiation [82]. The presence of methylated adenosine nucleotides in the form of N6-methyladenosine (m^6^A), which directly binds eIF3, has also been shown to drive the translation of circRNAs [81]. CircZNF609, which specifically controls myogenic cell proliferation, contains an open reading frame at the start codon, identical to the linear transcript, which terminates at an in-frame stop codon generated after cycling. CircZNF609 is associated with heavy multimers and is translated into protein in a splicing-dependent and cap-independent manner, providing an example of protein-encoding circRNAs in eukaryotes [83]. Zhang et al. found evidence that circSHPRH, a novel functional protein that translates SHPRH-146 A, acts as a tumor suppressor in glioblastoma [84].

### Application of CircRNAs

With a unique structure, stability, and immunogenicity, there has been an increased interest in the possible biological usefulness of circRNAs. CircRNAs are stable and with their translation capacity can be used as a complement to mRNA-based therapeutics using adeno-associated vector technology [85, 86]. Delivery of nano-formulations of circRNA containing IRES showed enhanced translational duration [85, 87]. The circRNA vaccine developed against SARS-CoV-2 showed durable antigen production and the capacity to produce neutralizing antibodies [88]. For the first time, the team of Wei Wensheng from Peking University prepared a circRNA vaccine against the delta variant of COVID-19. The vaccine had broad-spectrum protection against a variety of new coronavirus variants [88]. However, the means to improve production and delivery of circRNA and the means by which to reduce the cellular immune response triggered by circRNA are issues that require further development and refinement [89, 90].

Dysregulated circRNA expression is involved in a variety of diseases, including but not limited to autoimmune diseases, cancer, liver diseases, neurological diseases, cardiovascular diseases, and diabetes [64, 91–94]. There are many DNA, RNA and protein-based biomarkers currently used clinically as disease diagnostic aids [95]. CircRNAs are not easily degraded by nucleic acid exonucleases and therefore have a much longer half-life than linear RNAs, making circRNAs ideal candidates as biomarkers [96]. Significant expression levels of circRNAs have been found in blood, gastric juice, saliva, and urine from prostate cancer patients, suggesting that circRNAs have the potential to become biomarkers for early cancer diagnosis [43, 97–100]. In clinical studies, the combination of prostate specific antigen testing and circRNAs detection, greatly improved diagnosis of prostate cancer [101].

CircRNAs are also promising clinical therapeutic targets for a number of diseases. For example, F-circRNA aids acute promyelocytic leukemia cell transformation, cell survival, and resistance to treatment. As such it is a potential therapeutic target for disease treatment [102]. CircSLC8A1 is abundant within the heart, with inhibition of circSLC8A1 in vivo decreasing myocardial ischemia-reperfusion injury and stress overload-induced cardiac hypertrophy [103, 104]. However, the development of circRNA therapies is still in its infancy and there are a number of issues that require further improvement. Common issues are design and optimization of circRNAs overexpression vectors, improved cyclization, development of efficient delivery systems, improved chemical manufacturing procedures, and control process development [62]. There is much to be learned regarding circRNAs but they will likely become a powerful weapon for clinical treatment and diagnosis in the near future.

## Expression and biological function of CircRNAs in urinary system tumors

### Expression and biological function of CircRNAs in kidney Cancer

**Fig. 2:**
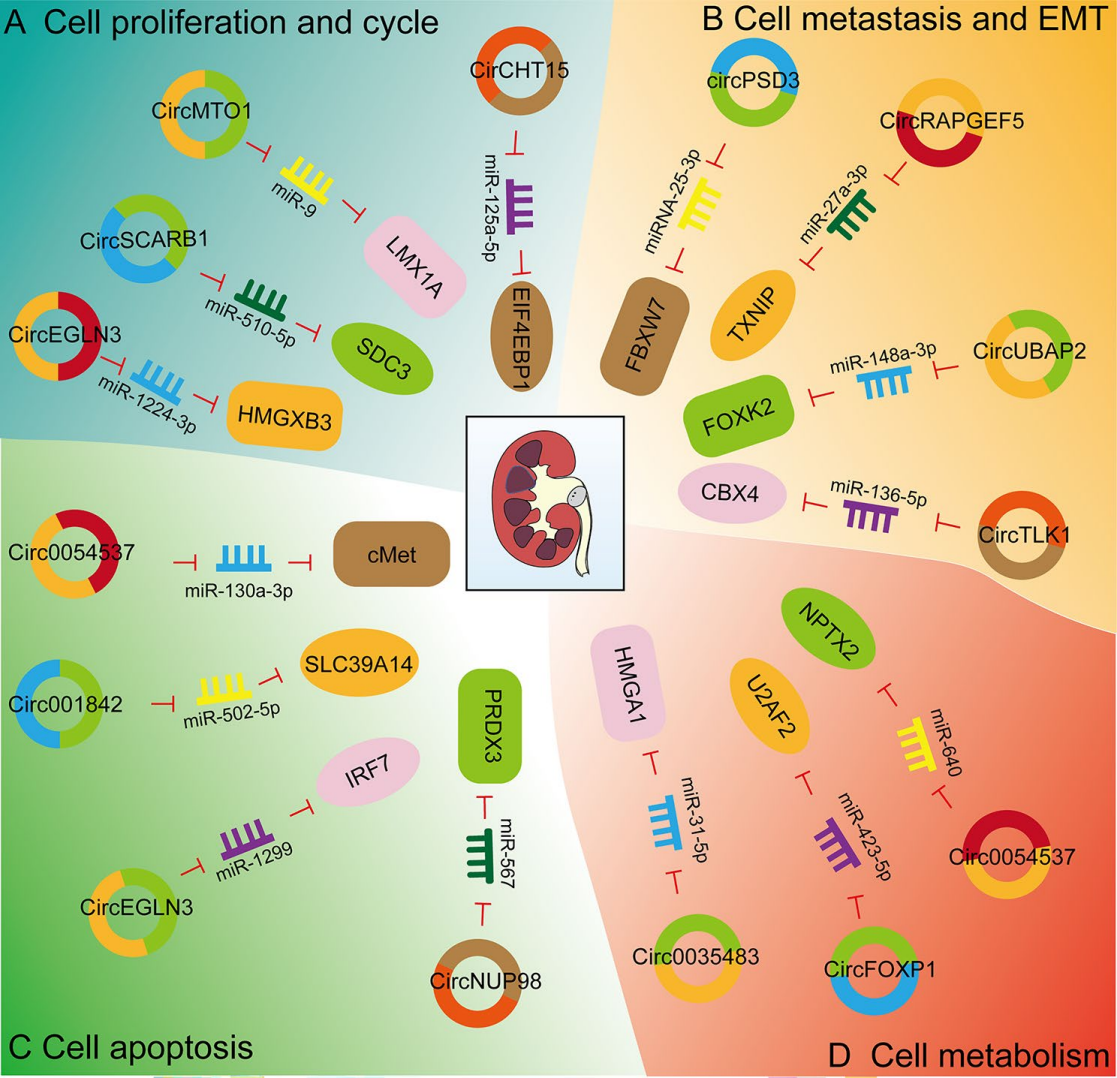
Biological functions of circRNAs in kidney cancer (A) CircRNAs are involved in the cell proliferation and cycle of kidney cancer. CircCHT15, circSCARB1, circMTO1, and circEGLN3 affect the cell proliferation of kidney cancer cells by regulating the expression of miRNAs and related proteins. (B) CircRNAs are involved in the metastasis and EMT of kidney cancer. CircPSD3, circRAPGEF5, circUBAP2, and circTLK1 affect the cell metastasis and EMT of kidney cancer cells by regulating the expression of miRNAs and related proteins. (C) CircRNAs are involved in the apoptosis of kidney cancer. Circ0054537, circ001842, circEGLN3, and circNUP98 affect the cell apoptosis of kidney cancer cells by regulating the expression of miRNAs and related proteins. (D) CircRNAs are involved in the cell metabolism of kidney cancer. Circ0035483, circFOXP1, and circ0054537 affect the cell metabolism of kidney cancer cells by regulating the expression of miRNAs and related proteins

CircRNAs play a critical role in the development and progression of kidney cancer. One study identified approximately 2000 differentially expressed circRNAs in kidney cancer cells using RNA-seq [105]. CircRNAs are involved in kidney cell proliferation, apoptosis, cell metastasis, invasion, epithelial-mesenchymal transition (EMT), cell metabolism, and can be used for patient prognosis (Fig. 2). In this section, we will describe the role of circRNAs in regulation of these cellular processes and will highlight the involved molecular mechanisms (Table 2).

#### Proliferation and cell cycle

Studies have shown that abnormal expression of circRNAs is associated with excessive renal cancer cell proliferation and disruption of cell cycle control. High expression levels of circ000926 in RCC are associated with a poor patient prognosis [106]. Silencing of circ000926 inhibits proliferation of RCC cells and inhibits the growth of xenografted tumors. Circ000926, a miRNA sponge, directly inhibits miR-411 and up-regulates CDH2. CircEGLN3 is highly expressed in kidney cancer, with inhibition of circEGLN3 reducing the proliferative ability of RCC ACHN and 769-P cell lines [107]. CircEGNL3 acts as a sponge for miR-1224-3p, which targets HMGXB3. CircEGNL3 indirectly up-regulates HMGXB3 by targeting miR-1224-3p, with overexpression of circEGLN3 reversing the inhibitory effect of miR-1224-3p on RCC. That study found circSCARB1 to be elevated in 30 pairs of RCC tissues and multiple RCC cell lines [108]. Inhibition of cell proliferation by silencing circSCARB1 is mediated by a direct interaction between circSCARB1 and miR-510-5p. SDC3 is a target gene of miR-510-5p. Transfection of miR-510-5p mimics not only suppresses SDC3 expression but also cell proliferation, with SDC3 co-transfection partially restoring cell proliferation control. Furthermore, overexpression of circMTO1 inhibits the proliferation of A498 and 786-O renal cancer cells, while circMTO1 silencing promotes the progression of SN12C and OS-RC-2 renal cancer cells [109]. CircMTO1 acts as a sponge for miR-9 and miR-223, suppressing their levels. Silencing circMTO1 downregulates LMX1A, a target of miR-9, promoting RCC cell proliferation. Transfection of a miR-9 mimic inhibited LMX1A expression, which confirmed that miR-9 exerts its function in RCC by regulation of LMX1A expression. Overexpression of a miR-9 inhibitor, with LMX1A, blocked the tumor-promoting effect of circMTO1 silencing. Similarly, high-throughput sequencing of circRNA in RCC cell lines found circTLK1 to be a novel candidate circRNA derived from the TLK1 gene [110]. CircTLK1 is highly expressed in RCC, with silencing of circTLK1 inhibiting the proliferation of RCC cells.

High expression levels of circSDHC in RCC tissues were found by GSE100186 and GSE137836 sequencing to be associated with advanced TNM stage and poor survival of RCC patients [111]. CircSDHC promotes tumor cell proliferation both in vivo and in vitro. RNA-pulldown and luciferase reporter assays demonstrated circSDHC to compete with miR-127-3p for binding, preventing inhibition of downstream CDKN3 and E2F1 pathways, resulting in malignant progression of RCC. Knockdown of circSDHC reduces CDKN3 expression, which inhibits the E2F1 pathway and can be rescued by miR-127-3p inhibitor therapy. CirCHT15 has been reported to be an independent prognostic indicator of overall survival and progression-free survival after surgical resection of ccRCC patients [112]. CirCHT15 is highly expressed in ccRCC tissues and renal cancer cell lines. In vivo and in vitro data suggest that cirCHT15 promotes the proliferation of ccRCC cells. CirCHT15 directly interacts with miR-125a-5p and regulates the expression of EIF4EBP1. CircDVL1 is poorly expressed in serum and tissues of ccRCC patients and is negatively related to malignant features of ccRCC [113]. Overexpression of circDVL1 inhibits the proliferation of various ccRCC cells and induces G1/S phase arrest. CircDVL1 acts as a molecular sponge for oncogenic miR-412-3p, preventing miR-412-3p-mediated repression of PCDH7 in ccRCC cells.

#### Metastasis and EMT

Cancer cell invasion and metastasis play a very important role in the occurrence and development of renal cancer. CircRNAs have been shown to regulate this process. For example, downregulation of circPSD3 in ccRCC tissues suggests that low levels of circPSD3 are associated with tumor metastasis in ccRCC patients [114]. CircPSD3 significantly inhibited cell migration, invasion, and EMT in vitro and blocked lung metastasis in vivo. CircPSD3 binds to miRNA-25-3p to regulate the expression of FBXW7. CircPSD3 inhibits tumor metastasis by suppressing the miR-25-3p/FBXW7-EMT axis and is expected to be a potential diagnostic and therapeutic target for ccRCC. The role of circRAPGEF5 in RCC is unclear, but analysis of samples from 245 RCC patients revealed that down-regulation of circRAPGEF5 was positively associated with aggressive clinical features [115]. We found that circRAPGEF5 inhibits the development and progression of RCC through the circRAPGEF5/miR-27a-3p/TXNIP pathway. A study showed that circ0001368 was downregulated in 786-O, ACHN, and A498 cell lines, as well as in RCC tissues [116]. That study found miR-492 to target miRNA of circ0001368. Inhibition of miR-492 inhibited cell metastasis, whereas overexpression of miR-492 enhanced cell metastasis. MiR-492 regulates LATS2 expression by binding to the 3′UTR region of LATS2. Previous studies have found that LATS2 inhibits HCC through the EZH2/DNMT1 axis and also inhibits glioma cell metastasis by up-regulation of tafazzin. Overexpression of LATS2 significantly inhibits the proliferation and invasion of ACHN and 786-O cells. Circ0001368 upregulates LATS2 expression by secreting miR-492, which inhibits renal cancer cell growth and invasion. Further, the circSCARB1/miR-510-5p/SDC3 signaling axis promotes invasion and migration of renal cancer cells [108]. CircUBAP2 expression is significantly downregulated in ccRCC tissues and cell lines [117]. Overexpression of circUBAP2 significantly inhibits the migration and invasion of ccRCC cells. CircUBAP2 targets miR-148a-3p, and miR-148a-3p reverses the inhibitory effect of circUBAP2 on ccRCC cell migration and invasion. Furthermore, FOXK2 was found to be a target gene of miR-148a-3p, with knockdown of FOXK2 reversing the inhibitory effect of the miR-148a-3p inhibitor on ccRCC cells. Further, circUBAP2 exerts a novel tumor-suppressive effect in ccRCC by regulating the miR-148a-3p/FOXK2 axis. Xia et al. found that circAKT3 was stably downregulated in ccRCC tissue samples by sequencing 60 ccRCC tissues and adjacent normal tissues as well as ccRCC cell lines [118]. Knockdown of circAKT3 promoted ccRCC migration and invasion, while overexpression of circAKT3 suppressed ccRCC metastasis. CircAKT3/miR-296-3p/E-cadherin is associated with the inhibition of ccRCC metastasis by circAKT3.

One study found circTLK1 to be mainly distributed within the cytoplasm, with high levels of expression positively related to distant metastasis and poor patient prognosis. Expression of CBX4 is positively regulated by miR-136-5p sponging [110]. Enhanced CBX4 expression reverses the phenotypic suppression of RCC cells induced by circTLK1 inhibition. Additionally, in RCC tissues CBX4 expression is positively related to VEGFA expression. CBX4 knockout significantly inhibits the expression of VEGFA in RCC cells. Another study found that circESRP1 was poorly expressed in renal cancer tissues and renal cancer cells, and its expression level was negatively related to advanced tumor size, TNM stage, and distant metastasis of ccRCC [119]. CircESRP1 competitively binds to miR-394. A downstream target of miR-394 is CTCF, which specifically promotes circESRP1 transcription, regulating the circESRP1/miR-3942 pathway and forming a positive feedback loop. This process regulates ccRCC cell function through a c-Myc-mediated EMT mechanism. CircMYLK is significantly up-regulated in RCC, with circMYLK binding miR-513a-5p and promoting the expression of VEGFC, which promotes the tumorigenesis of RCC cells [120]. Silencing circMYLK inhibits RCC metastasis in vitro and in vivo. Overexpression of circTXNDC11 is associated with advanced TNM staging and lymph node metastasis in renal cancer [121]. Knockdown of circTXNDC11 inhibits cell invasion and metastasis in vitro. CircTXNDC11 promotes RCC migration and invasion by activating the MAPK/ERK pathway.

#### Apoptosis

CircRNAs have been reported to regulate apoptosis in renal cancer cells. MiR-130a-3p is expressed at abnormally low levels in RCC cells, with up-regulation of miR-130a-3p promoting apoptosis of RCC cells [122]. A direct interaction between circ0054537 and miR-130a-3p was found by RNA-pulldown and luciferase reporter assay. The oncogene c-Met can be targeted by miR-130a-3p and is jointly controlled by circ0054537 and miR-130a-3p, affecting the tumorigenesis and development of RCC. Similarly, high expression levels of circSCARB1 are found in renal cancer cells with knockdown of circSCARB1 promoting apoptosis of 786-O and A498 cell lines [108]. Circ0005875 is up-regulated in RCC tissues and cells, with knockdown of the circ0005875 gene increasing the expression of miR-502-5p, thus promoting the apoptosis of renal cancer cells [123]. Sequencing results identified elevated circEGLN3 in RCC tissues and cell lines and predicted a poor prognosis for RCC patients [124]. Silencing of circEGLN3 promotes apoptosis in RCC cells. CircEGLN3 is primarily located in the cytoplasm where it inhibits RCC progression by targeting miR-1299, which regulates the expression of IRF7. In addition, circ001842 is highly expressed in RCC. Circ001842 is involved in RCC pathogenesis through a miR-502-5p-dependent SLC39A14 mechanism [125]. Knockdown of circ001842 promotes apoptosis of RCC cells. Another study found that circNUP98 was selectively up-regulated in 78 pairs RCC tissues, when compared to adjacent normal tissues. Silencing of circNUP98 down-regulated PRDX3 by up-regulation of miR-567, thereby promoting the apoptosis of RCC and inhibiting the progression of RCC [126]. Further, STAT3 has been identified as an inducer of circNUP98 in RCC cells. CircNUP98 functions as an oncogene through a novel STAT3/circNUP98/miR-567/PRDX3 axis, providing a potential biomarker and therapeutic target for RCC therapy.

#### Metabolism

CirCRNAs have been shown to control kidney cancer cell metabolism. For example, circ0035483 is upregulated in RCC tissues and cells. Downregulation of circ0035483 suppresses glucose consumption and lactate production in RCC cells, suggesting that overexpression of circ0035483 promotes glycolytic metabolism in RCC cells [127]. MiR-31-5p up-regulation is associated with circ0035483 knockout in RCC cells. MiR-31-5p targets HMGA1 and suppresses the malignant behavior of RCC cells by negative regulation of HMGA1. Moreover, circFOXP1 is highly expressed in RCC tissues and cells, with down-regulation of circFOXP1 inhibiting the glycolysis of renal cancer cells [128]. ZNF263, upstream of circFOXP1, promotes RCC progression through the miR-423-5p/U2AF2 axis. That study found circ0054537 to be distributed within the cytoplasm and to be up-regulated in RCC tissues and cells [129]. Circ0054537 gene knockout inhibits glycolysis in RCC cells but promotes apoptosis. Luciferase reporter assay and RNA-pulldown confirmed that circ0054537 binds miR-640 and targets NPTX2.

**Table 2:** Overview of deregulated circRNAs in renal cell carcinoma

CircRNA	Expression	Function	Target microRNA	miRNA target genes/protein	Ref
Circ000926	Up	Proliferation(+) Metastasis(+)	miR-411	CDH2	[106]
CircEGLN3	Up	Proliferation(+) Metastasis(+)	miR-1224-3p	HMGXB3	[107]
CircRAPGEF5	Down	Proliferation(-) Metastasis(-)	miR-27a-3p	TXNIP	[115]
Circ0001368	Down	Proliferation(-) Metastasis(-)	miR-492	LATS2	[116]
Circ0054537	Up	Proliferation(+) Metastasis(+) Apoptosis(−)	miR- 130a-3p	cMet	[122]
CircSCARB1	Up	Proliferation(+) Metastasis(+) Apoptosis(−)	miR-510-5p	SDC3	[108]
CircUBAP2	Down	Proliferation(-) Metastasis(-)	miR-148a-3p	FOXK2	[117]
CircMTO1	Down	Proliferation(-) Metastasis(-)	miR-9	LMX1A	[109]
CircAKT3	Down	Metastasis(-)	miR-296-3p	E-cadherin	[118]
CircTLK1	Up	Proliferation(+) Metastasis(+)	miR-136-5p	CBX4	[110]
CircSDHC	Up	Proliferation(+) Metastasis(+)	miR-127-3p	CDKN3/E2F1	[111]
CircCHST15	Up	Proliferation(+) Metastasis(+)	miR-125a-5p	EIF4EBP1	[112]
CircESRP1	Down	Proliferation(-) Metastasis(-)	miR-3942	CTCF	[119]
Circ0005875	Up	Proliferation(+) Metastasis(+) Apoptosis(−)	miR-502-5p	ETS1	[123]
Circ0035483	Up	Proliferation(+) Metastasis(+)	miR-31-5p	HMGA1	[127]
CircDVL1	Down	Proliferation(-) Metastasis(-) Apoptosis(+)	miR-412-3p	PCDH7	[113]
CircEGLN3	Up	Proliferation(+) Metastasis(+) Apoptosis(−)	miR-1299	IRF7	[124]
CircMYLK	Up	Proliferation(+) Metastasis(+)	miR-513a-5p	VEGFC	[120]
Circ001842	Up	Proliferation(+) Metastasis(+) Apoptosis(−)	miR-502-5p	SLC39A14	[125]
CircNUP98	Up	Proliferation(+) Metastasis(+) Apoptosis(−)	miR-567	PRDX3	[126]
CircFOXP1	Up	Proliferation(+) Metastasis(+)	miR-423-5p	U2AF2	[128]
CircPSD3	Down	Proliferation(-) Metastasis(-)	miR-25-3p	FBXW7	[114]
CircTXNDC11	Up	Metastasis(+)	-	MAPK/ERK	[121]
Circ0054537	UP	Glycolysis(+) Apoptosis(−)	miR-640	NPTX2	[129]

### Expression and biological function of CircRNAs in bladder Cancer

**Fig. 3:**
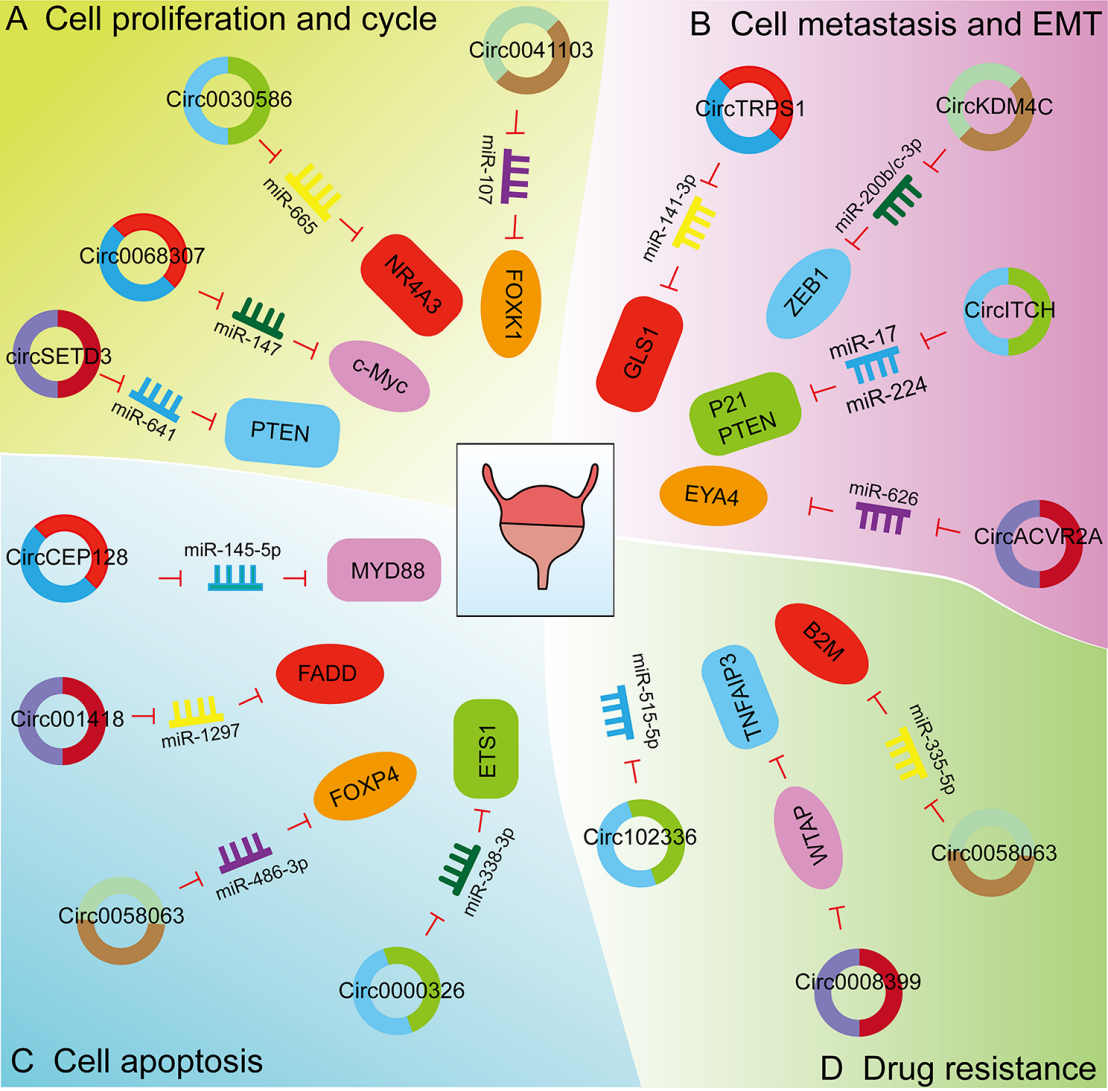
Biological functions of circRNAs in bladder cancer (BCa) (A) CircRNAs are involved in the cell proliferation and cycle of BCa. CircSETD3, circ0068307, circ0030586, and circ0041103 affect the cell proliferation of BCa cells by regulating the expression of miRNAs and related proteins. (B) CircRNAs are involved in the metastasis and EMT of BCa. CircTRPS1, circKDM4C, circITCH, and circACVR2A affect the cell metastasis and EMT of BCa cells by regulating the expression of miRNAs and related proteins. (C) CircRNAs are involved in the apoptosis of BCa. CircCEP128, circ001418, circ0058063, and circ0000326 affect the cell apoptosis of BCa cells by regulating the expression of miRNAs and related proteins. (D) CircRNAs are involved in the drug resistance of BCa. Circ102336, circ0008399, and circ0058063 affect the drug resistance of BCa cells by regulating the expression of miRNAs and related proteins

Aberrant expression of circRNAs is associated with the development of BCa. Several high-throughput experiments have shown that circRNAs expression profiles are dysregulated in BCa [130]. CircRNAs are associated with patient prognosis, cell proliferation, apoptosis, cell metastasis, invasion, EMT, and drug resistance (Fig. 3). In this section, we will describe the role of circRNAs in regulating these cellular processes and will highlight circRNA molecular mechanisms (Table 3).

#### Proliferation and the cell cycle

CircRNAs play an important regulatory role in the malignant behavior of BCa cells. Circ0030586 is localized to the cytoplasm where it is poorly expressed in BCa tissues and cells [131]. Overexpression of circ0030586 inhibits BCa cell proliferation and stemness in vitro and also inhibits tumor growth in vivo. Circ0030586 binds miR-665, which is highly expressed in BCa tissues and cells, while NR4A3 is down-regulated in BCa. MiR-665 overexpression or NR4A3 silencing reverses the inhibitory effects of circ0030586 overexpression on BCa cell proliferation and stemness. The level of circ0091017 expression is significantly down-regulated in BCa tissues and cell lines, while the expression of miR-589-5p is up-regulated [132]. In vitro studies found that overexpression of circ0091017 inhibits the proliferation of BCa cells and the expression of miRNA-589-5p. However, overexpression of miR-589-5p reverses the inhibitory effect of circ0091017 on the malignant phenotype of BCa cells. Circ0041103 is aberrantly upregulated in BCa tissues and cell lines [133]. Knockdown of circ0041103 inhibits the proliferation and metastasis of T24 and UM-UC-3 cells. MiR-107 is the binding target of circ0041103, with FOXK1 a downstream gene target of miR-107. Overexpression of circ0041103 reverses the suppressed proliferation and metastatic capacity of T24 and UM-UC-3 cells that overexpress miR-107. Circ0068307 is highly expressed in BCa cell lines, with silencing of circ0068307 resulting in reduced cancer stem cell differentiation by up-regulation of miR-147 expression [134]. The transcription of c-Myc is inhibited after miR-147 activation. MiR-147 down-regulation of the CSC-related proteins (OCT-4, NANOG, and Sox-2) results in decreased cell proliferation and migration. One study found that the expression of circ0001944 was increased in BCa tissues and related to a poor prognosis for BCa patients. Downregulation of circ0001944 reduced BCa invasion and metastasis in vivo and in vitro [135]. Circ0001944 expression promotes BCa progression by adsorbing miR-548 and enhancing PROK2 expression. Another study found that circUBE2K was located on chromosome 4, with high expression of circUBE2K associated with a poor prognosis in BCa patients [136]. In vitro studies found that down-regulation of circUBE2K reduced the proliferative capacity of BCa cells. By subcutaneous xenograft, circUBE2K was found to significantly increase the proliferation of BCa cells in vivo. Qi et al. found that circ100984 was highly expressed in BCa tissues, with knockdown of circ100984 inhibiting the growth of BCa tumors [137]. Circ100984 adsorbs miR-432-3p and indirectly regulates YBX-1 and EMT-related molecules. YBX-1 and c-Jun act as transcriptional regulators of β-catenin and YBX-1, respectively, in BCa cells. Knockdown of YBX-1 suppresses the expression of β-catenin and c-Jun, whereas down-regulation of c-Jun conversely inhibits the expression of YBX-1 and β-catenin. Mei et al. found that circSETD3, a tumor suppressor, inhibits the proliferation of BCa cells [138]. CircSETD3 is significantly down-regulated in cancerous clinical tissues and cell lines, and is often deficient in BCa patients with larger tumors, advanced clinical stage, positive lymph node metastasis, and poor prognosis. We found that the circSETD3/miR-641/PTEN axis regulates the malignant phenotype of BCa cells in vitro.

#### Metastasis and EMT

A number of circRNAs contribute to metastasis and EMT. CircTRPS1 is significantly increased in exosomes derived from BCa tissue as well as from urine and serum of patients [130]. Metabolomics and RNA-seq have demonstrated circTRPS1 to enhance the function of GLS1, a key enzyme for glutamine metabolism. Overexpression of GLS1 enhances both proliferation and invasiveness of BCa cells and impairs anti-tumor immunity within the BCa microenvironment. Therefore, circTRPS1 may be a novel biomarker and therapeutic target for BCa patients. Circ0006948 is upregulated in BCa tissues and cell lines [139]. Increased expression of cir0006948 is related to advanced tumor stage, lymph node metastasis, high distance transfer rate, and poor prognosis. Knockdown of circ0006948 reduces the proliferation and metastatic capacity of BCa cells, resulting in upregulation of E-cadherin and downregulation of N-cadherin, Vimentin, β-catenin, and MMP-9. CircITCH was found to be poorly expressed in BCa tissues and cell lines, with circITCH expression inhibiting cell proliferation, migration, invasion, and metastasis [140]. CircITCH up-regulates the expression of miR-17 and miR-224 that target p21 and PTEN, respectively, which reduce the invasiveness of BCa. CircACVR2A is poorly expressed in BCa tissues and cell lines [141]. Reductions in circACVR2A are positively related to an aggressive clinical pathology. CircACVR2A is an independent risk factor for overall survival rate in patients with BCa. CircACVR2A directly interacts with miR-626, functioning as a miRNA sponge, which regulates the expression of EYA4. One study found that circ0068871 was overexpressed in BCa tissues and cell lines, whereas miR-181a-5p expression was inhibited [142]. Deletion of circ0068871 or up-regulation of miR-181a-5p inhibited BCa cell proliferation and migration in vitro and in vivo. Circ0068871 up-regulates FGFR3 expression and activates STAT3 to promote BCa progression by targeting miR-181a-5p. Hou et al. found that circ0008532 expression was significantly up-regulated in BCa tissues and cell lines and positively related to BCa progression by secreting miR-155-5p/miR-330-5p, which affects MTGR1 expression and Notch signaling activity [143]. Circ0008532 knockdown suppresses the invasion of BCa cells. Zhao et al. found that circ0000658 was highly expressed in BCa tissue samples and cell lines and related to a poor prognosis for BCa patients [144]. Circ0000658 binds competitively to miR-498, thereby limiting miR-498 expression. Further, circ0000658 weakens binding of miR-498 to the target gene, HMGA2, which up-regulates the expression of HMGA2. Overexpression of circ0000658 results in decreased expression of E-cadherin and increased expression of N-cadherin, snail, slug, ZEB1 and Twist in BCa cells. Estrogen receptor α (ERα) plays a protective role in BCa by reducing BCa invasion through reduced expression of circ0023642 [145]. ERα decreases circ0023642 levels and subsequently increases miR-490-5p expression, resulting in decreased EGFR expression, thereby inhibiting BCa cell invasion. Another study found that circST6GALNAC6 expression was down-regulated in BCa tissues and cells [146]. Overexpression of circST6GALNAC6 effectively inhibited cell proliferation, migration, invasion, and EMT in vitro and inhibited BCa metastasis in vivo. Binding of the transcription factor, SP1, to circST6GALNAC6 mRNA transcripts activates circST6GALNAC6 transcription. CircKDM4C is derived from the KDM4C gene and is highly expressed in BCa cell lines and tissues. Overexpression of circKDM4C significantly enhances the migration and invasion of BCa cells, while silencing of circKDM4C inhibits migration and invasion of BCa cells [147]. CircKDM4C can interact with miR-200b-3p and miR-200c-3p, thereby enhancing ZEB1 expression and promoting EMT.

#### Apoptosis

Apoptosis is a normal physiological process that is usually inhibited in tumor tissues. Some circRNAs can promote apoptosis. Circ001418 expression is increased in BCa patients [148]. In vitro studies found that knockdown of circ001418 inhibited cell proliferation and induced apoptosis. By inhibiting miR-1297, circ001418 overexpression increases the expression of EphA2 and cytochrome c proteins while suppressing the expression of FADD protein. CircST6GALNAC6 binds miR-200a-3p to regulate STMN1 [146]. STMN1 is involved in BCa EMT, with metastasis regulated by the circST6GALNAC6/miR-200a-3p axis. Further, circRNA 0058063 was found to be aberrantly expressed in different BCa cell lines and BCa cancer tissues [149]. Knockdown of circ0058063 significantly reduced cell proliferation and invasion and promoted apoptosis. Circ0058063 and FOXP4 directly bind miR-486-3p, indicating that circ0058063 regulates FOXP4 expression by competitively binding miR-486-3p. That study found circ0000629 to be expressed in the cytoplasm and to inhibit the development and metastasis of BCa cells [150]. By enhancing the expression of BAX and PUMA, BCL-2 expression was inhibited, promoting cellular apoptosis. Circ0000629 binds miR-1290 to regulate the expression of CDC73. Overexpression of circ0000629 reduces BCa tumor growth in vivo. The circCEP128/miR-145-5p/MYD88 axis plays an important role in BCa [151]. Knockdown of circCEP128 reduces cell viability and migration and induces cell cycle arrest, promoting apoptosis of BCa cells. Overexpression of miR-145-5p or knockdown of circCEP128 promotes the expression of the MAPK signaling pathway and related proteins. Furthermore, knockdown of circCEP128 inhibits the growth of BCa tissue in vivo. CircCEP128 overexpression promotes BCa progression by regulation of miR-145-5p and MYD88 through MAPK signaling. Circ0000326 expression is significantly elevated in BCa cell lines and tissues, with circ0000326 promoting BCa cell growth and migration as well as inhibiting apoptosis [152]. Circ0000326 and ETS1 bind directly to miR-338-3p. In addition, circ0000326 binds miR-338-3p and up-regulates ETS1 expression. Circ0000326 also activates the PI3K/AKT pathway through the miR-338-3p/ETS1 axis.

#### Drug resistance

Acquired drug resistance is a significant impediment to tumor therapy. Some cirRNAs are capable of reversing tumor drug resistance. In BCa tissue samples and cell lines, circ102336 is highly expressed and relates to poor overall survival of BCa patients [153]. Circ102336 knockdown increases sensitivity of T24 and 5637 cell lines for CDDP. Circ102336 directly binds and negatively regulates miR-515-5p. EIF4A3 up-regulates circ0008399 in BCa tissues and cells [154]. Circ0008399 binds WTAP to promote the formation of the WTAP/METTL3/METTL14 m^6^A methyltransferase complex. Circ0008399 increases the expression of TNFAIP3 by increasing mRNA stability in an m^6^A-dependent manner. Activation of the circ0008399/WTAP/TNFAIP3 pathway reduces the sensitivity of BCa to cisplatin, with targeting of the circ0008399/WTAP/TNFAIP3 axis enhancing the efficacy of cisplatin. Circ0058063 is highly expressed in CDDP-resistant BCa tissues and cells [155]. Knockdown of circ0058063 inhibits cell proliferation and tumor growth, while inducing apoptosis in CDDP-resistant BCa cells in vitro and in vivo. Circ0058063 promotes CDDP resistance in BCa cells by secreting miR-335-5p, which up-regulates B2M levels by promoting the expression of genes associated with cancer stem cell properties. In addition, miR-335-5p inhibition reverses inhibition of cell proliferation and promotes apoptosis by silencing circ0058063.

**Table 3:** Overview of deregulated circRNAs in bladder cancer

CircRNA	Expression	Function	Target microRNA	miRNA target genes/protein	Ref
Circ0030586	Down	Proliferation(-)	miR-665	NR4A3	[131]
Circ102336	Up	Proliferation(+) Resistance(-)	miR-515-5p	-	[153]
CircTRPS1	Up	Proliferation(+) Metastasis(+)	miR-141-3p	GLS1	[130]
Circ001418	Up	Proliferation(+) Metastasis(+) Apoptosis(−)	miR-1297	EphA2	[148]
Circ0091017	Up	Proliferation(+) Metastasis(+)	miR-589-5p	-	[132]
Circ0041103	Up	Proliferation(+) Metastasis(+)	miR-107	FOXK1	[133]
Circ0058063	Up	Proliferation(+) Apoptosis(−) Resistance(−)	miR-335-5p	B2M	[155]
Circ0068307	Up	Proliferation(+) Metastasis(+)	miR-147	c-Myc	[134]
CircITCH	Down	Proliferation(-) Metastasis(-) Apoptosis(+)	miR-17/ miR-224	p21/ PTEN	[140]
CircACVR2A	Down	Proliferation(-) Metastasis(-)	miR-626	EYA4	[141]
Circ0068871	Up	Proliferation(+) Metastasis(+) Apoptosis(−)	miR-181a-5p	STAT3	[142]
Circ0008532	Up	Metastasis(+)	miR-155-5p /miR-330-5p	MTGR1	[143]
Circ0001944	Up	Proliferation(+) Metastasis(+)	miR-548	PROK2	[135]
Circ0000658	Up	Metastasis(+)	miR-498	HMGA2	[144]
Circ0023642	Up	Metastasis(+)	miR-490-5p	EGFR	[145]
CircST6GALNAC6	Down	Proliferation(-) Metastasis(-)	miR-200a-3p	STMN1	[146]
CircUBE2K	Up	Proliferation(+) Metastasis(+)	miR-516b-5p	ARHGAP5	[136]
CircKDM4C	Up	Metastasis(+)	miR-200bc-3p	ZEB1	[147]
Circ0058063	Up	Metastasis(+) Apoptosis(−)	miR-486-3p	FOXP4	[149]
Circ100984	Up	Proliferation(+) Metastasis(+)	miR-432-3p	c-Jun/YBX-1/β-catenin	[137]
CircSETD3	Down	Proliferation(-) Metastasis(-)	miR-641	PTEN	[138]
Circ0006948	Up	Proliferation(+) Metastasis(+)	-	N-cadherin	[139]
Circ0000629	Down	Proliferation(-) Metastasis(-) Apoptosis(+)	miR-1290	CDC73	[150]
CircCEP128	Up	Proliferation(+) Metastasis(+) Apoptosis(−)	miR-145-5p	MYD88	[151]
Circ0000326	Up	Proliferation(+) Metastasis(+) Apoptosis(−)	miR-338-3p	ETS1	[152]
Circ0008399	Up	Resistance(+)	-	WTAP	[154]

### Expression and biological functions of CircRNAs in prostate Cancer

**Fig. 4:**
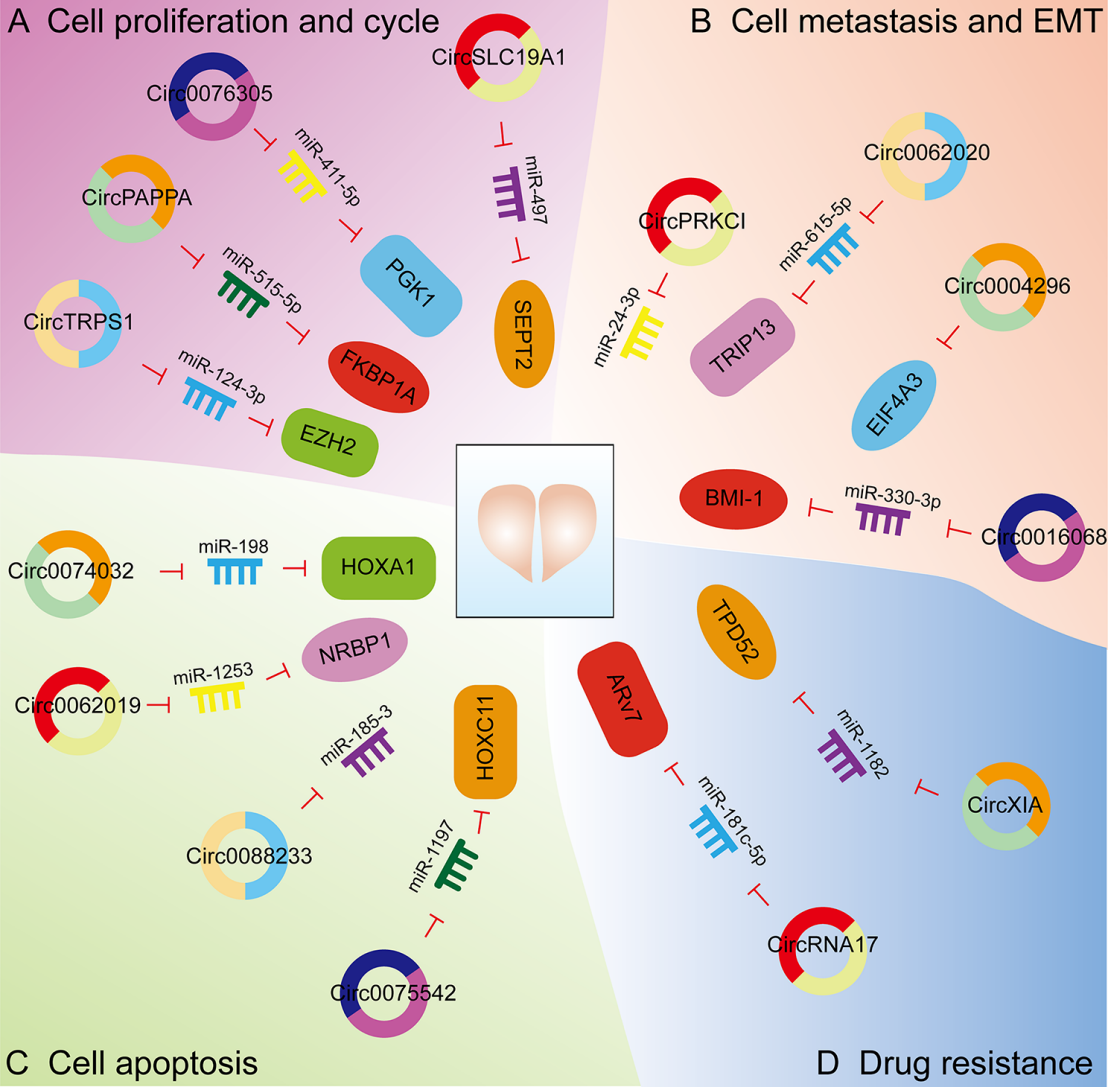
Biological functions of circRNAs in prostate cancer (PCa) (A) CircRNAs are involved in the cell proliferation and cycle of PCa. CircTRPS1, circPAPPA, circ0076305, and circSLC19A1 affect the cell proliferation of PCa cells by regulating the expression of miRNAs and related proteins. (B) CircRNAs are involved in the metastasis and EMT of PCa. CircPRKCI, circ0062020, circ0004296, and circ0016068 affect the cell metastasis and EMT of PCa cells by regulating the expression of miRNAs and related proteins. (C) CircRNAs are involved in the apoptosis of PCa. Circ0074032, circ0062019, circ0088233, and circ0075542 affect the cell apoptosis of PCa cells by regulating the expression of miRNAs and related proteins. (D) CircRNAs are involved in the drug resistance of PCa. CircRNA17 and circXIA affect the drug resistance of PCa cells by regulating the expression of miRNAs and related proteins

CircRNAs are involved in PCa cell proliferation, apoptosis, cell metastasis, invasion, EMT, and drug resistance. CircRNAs are also useful for patient prognosis (Fig. 4). In this section, we will describe the role of circRNAs in the regulation of PCa cellular processes and will highlight the involved circRNA molecular mechanisms (Table 4).

#### Proliferation and the cell cycle

PCa cells can maintain an active proliferative state by activation of cell proliferation signaling pathways. CirRNAs enhance this process. Circ0001206 is downregulated in PCa and significantly related to the clinical features of PCa patients [156]. ROC curves show circ0001206 to have good diagnostic value as a biomarker for PCa. Overexpression of circ0001206 inhibits PCa cell proliferation in vitro and prevents tumor growth in vivo. Dual-luciferase reporter gene assays demonstrated circ0001206 to bind to miR-1285-5p. Smad4 expression is increased by overexpression of circ0001206, and this effect is partially reversed by co-transfection with miR-1285-5p mimics. Similarly, the expression of circTRPS1 is increased in PCa patient samples and cell lines, with circTRPS1 acting as an oncogene with miR-124-3p, resulting in enhanced EZH2 expression and promotion of cancer stem-like cell-mediated PCa progression [157]. In vitro and in vivo experiments demonstrated circTRPS1 silencing to inhibit proliferation of PCa cells. CircPAPPA was found to be highly expressed in PCa tissues. Knockdown of circPAPPA effectively inhibited the proliferative capacity of PCa cell lines DU145 and LNCap, and also inhibited tumor metastasis by promoting apoptosis [158]. CircPAPPA binds and negatively relates to miR-515-5p. MiR-515-5p silencing greatly reduces circPAPPA knockdown-mediated effects in PCa cells. MiR-515-5p directly binds to FKBP1A, with the effects of miR-515-5p overexpression partially offset by FKBP1A overexpression. CircPAPPA silencing partially reduces FKBP1A protein levels by increasing miR-515-5p expression. CircPAPPA knockout also significantly inhibits tumor growth in vivo. In addition, circ0076305 is highly expressed in PCa [159]. Circ0076305 silencing inhibits cell growth and glycolysis while triggering apoptosis in PCa cells. Circ0076305 targets miR-411-5p, with miR-411-5p inhibition counteracting the effects of circ0076305 silencing in PCa cells. Furthermore, miR-411-5p directly targets PGK1, with miR-411-5p up-regulation suppressing PCa cell malignant behavior by decreasing PGK1. Lin et al. found increased expression of circSLC19A1 in PCa cells and PCa secreted extracellular vesicles (EVs) [160]. EVs with high expression of circSLC19A1 can be taken up by PCa cells and in that manner promote cell proliferation and invasion. CircSLC19A1 can directly bind to miR-497. In PCa cells, the expression of miR-497 is down-regulated, while the expression of its target, septin 2 (SEPT2), is significantly up-regulated. This up-regulation activates the ERK1/2 pathway and regulates the growth and invasion of PCa cells. These results suggest that the miR-497/SEPT2/ERK1/2 pathway, regulated by EV-derived circSLC19A1, is important in PCa growth and invasion.

A study found that circ0057558 was highly expressed in PCa tissues and cell lines [161]. Circ0057558 knockout in PCa cells significantly inhibited cell proliferation and colony formation but promoted cell arrest and enhanced sensitivity to doxorubicin. Circ0057558 can bind miR-206, which is negatively related to PCa tissue expression. Further, miR-206 targets USP33, with USP33 overexpression partially blocking the effects of miR-206 mimics on prostate cell proliferation. USP33 can bind to c-Myc. Increased c-Myc protein induced by circ0057558 overexpression partially reverses a miR-206 mimic. The proliferation-inhibitory activity of the c-Myc inhibitor 361 was pronounced in primary PCa cells and in patient-derived xenograft models with higher circ0057558 levels. In another study, circ0062019 was found to be highly expressed in PCa cell lines [162]. Silencing circ0062019 can effectively inhibit cell proliferation and metastasis. Circ0062019 binds miR-195-5p and miR-195-5p binds HMGA2. The circ0062019-induced malignant phenotype of PCa cells is reversed by the down-regulation of HMGA2. CircFMN2 expression is significantly elevated in PCa tissues [163]. Knockdown of circFMN2 suppresses cell proliferation and inhibits tumor growth in vitro and in vivo. CircFMN2 can act as a sponge for miR-1238, which up-regulates the expression of LHX2.

#### Metastasis and EMT

Blocking metastasis of PCa cells is critical to the inhibition of tumor progression. Circ102004 is expressed at greater levels in PCa samples than in paracancerous tissues [164]. Circ102004 exerts an oncogenic role in PCa by stimulating cancer cell migration and invasion. Overexpression of Circ102004 promotes PCa cell metastasis and overgrowth of xenografted tumors, as well as affects signaling pathways such as ERK, JNK, and Hedgehog. CircPRKCI was found to be up-regulated in PCa tissues and related to patient metastatic rate in an analysis of 40 PCa tissues and normal paracancerous tissues [165]. The metastatic capacity of DU-145 and PC-3 cell lines was inhibited after knockdown of circPRKCI, which binds miR-24-3p and exhibits a negative relationship with circPRKC. Likewise, circ0003258 was significantly up-regulated in PCa tissues and cells and related to PCa cell metastais [166]. High expression of circ0003258 promotes PCa cell migration by inducing EMT in vitro and tumor metastasis in vivo. Circ0003258 increases the expression of Rho GTPase activating protein 5 by binding to miR-653-5p. Moreover, circ0003258 binds to IGF2BP3 within the cytoplasm, enhancing stability of HDAC4 mRNA, which activates the ERK signaling pathway and triggers EMT, ultimately accelerating PCa metastasis.

Circ0004296 expression is reduced in PCa tissues, patient blood, as well as urine, with a negative relationship to metastasis [167]. In vitro and in vivo experiments demonstrated circ0004296 to inhibit PCa cell proliferation, migration, invasion, and EMT. Circ0004296 is expressed in the nucleus and interacts with the RNA-binding protein, EIF4A3. The expression of EIF4A3 is significantly up-regulated in PCa tissues and relates to PCa cell metastasis. Silencing of EIF4A3 inhibits PCa cell proliferation, migration, invasion, and EMT. Cai et al. found that circ0062020 was up-regulated in PCa tissues and cells, especially in radiation-hardened PCa tissues [168]. Further, circ0062020 knockdown enhanced radio-sensitivity by modulation of the miR-615-5p/thyroid hormone receptor interactor 13 (TRIP13) axis in PCa cells. Furthermore, circ0062020 knockout inhibited PCa tumor growth in vivo. These findings may contribute to improvements in PCa therapy. Circ0016068 is highly expressed in PCa tissues and cell lines [169]. Circ0016068 promotes PCa cell EMT, growth, migration, and invasion in vitro, as well as tumor growth and metastasis in a mouse model of PCa. Circ0016068 competes with B lymphoma Moloney mouse leukemia virus insertion region-1 (BMI-1) for binding to miR-330-3p. Circ0016068 sequesters miR-330-3p and releases BMI-1 to enhance PCa cell proliferation, migration, and invasion, as well as xenograft tumor metastasis. Circ0030586 was found to be highly expressed in PCa cells using the GEO database [170]. Knockdown of circ0030586 in PC3 cells inhibited PCa cell proliferation, migration and invasion, and resulted in significant up-regulation of E-cadherin and significant down-regulation of p-AKT/AKT, IKKα, PIK3CB, and Twist. Circ0001686 expression was up-regulated in PCa cells, while miR-411-5p expression was down-regulated [171]. Furthermore, circ0001686 promoted cell proliferation, migration and invasion. Circ0001686 decreased miR-411-5p levels, affecting the downstream target genes, SMAD3 and TGFBR2.

#### Apoptosis

Promotion of PCa apoptosis may contribute to better outcomes for PCa patients. Circ0074032 is overexpressed in PCa tissues, with high expression levels of circ0074032 associated with a poor prognosis for PCa patients [172]. Circ0074032 silencing reduces xenograft tumor growth in vivo and induces apoptosis and inhibition of PCa cell proliferation. Circ0074032 binds miR-198, with miR-198 inhibition abrogating the effects of circ0074032 on silencing-mediated PCa cell proliferation and apoptosis. Further, miR-198 directly targets homeobox A1, which attenuates miR-198 mimetic-mediated effects on the malignant phenotype of PCa cells.

A study found that circ0062019 is highly expressed in PCa tissue and multiple PCa cell lines [173]. Knockdown of circ0062019 inhibits the proliferation and the cell cycle of PCa cells, promoting cellular apoptosis. Inhibition of PCNA and BCL-2 enhances the expression of BAX. Circ0062019 promotes PCa cell progression by binding miR-1253. MiR-1253 impedes PCa cell progression by regulation of NRBP1. By analysis of 46 pairs of PCa and adjacent normal tissues, circ0088233 was found to be up-regulated and its expression level related to TNM stage [174]. Knockdown of circ0088233 reduced cell proliferation, migration, and invasion, as well as induced G1 phase arrest and apoptosis. Circ0088233 binds miR-185-3, with expression levels negatively related. Circ0088233 overexpression blocked the effects of miR-185-3p on cell proliferation, migration, invasion, cell cycle progression, and apoptosis. Huang et al. found that circ0075542 expression was down-regulated in prostate tumor tissues [175]. Overexpression of circ0075542 inhibited cell proliferation, decreased migration, decreased invasion, and promoted apoptosis. Circ0075542 targeted HOXC11 via adsorption of miR-1197. Circ0057553 is significantly up-regulated in PCa tissues and cells [176]. Knockdown of circ0057553 inhibits PCa cell viability, migration, invasion, and glycolysis, while promoting apoptosis. Circ0057553 binds to miR-515-5p and miR-515-5p, targeting YES1. MiR-515-5p inhibition partially rescues the function of circ0057553 knockdown, while YES1 restores the effect of miR-515-5p overexpression. Further, circ0006404 is highly expressed in PCa patients [177]. Circ0006404 promotes PCa cell survival, metastasis, and proliferation, as well as inhibits PCa cellular apoptosis. Circ0006404 directly targets miR-1299, with silencing of miR-1299 reversing the effects of circ0006404 interference on PCa cells. CFL2 binds directly to miR-1299, with effects induced by miR-1299 in PCa cells largely attenuated by CFL2 overexpression. CFL2 is regulated by the circ0006404/miR-1299 axis in PCa cells. Circ0006404 promotes PCa progression through the miR-1299/CFL2 axis in vivo. In an assessment of 60 patients and PC cell lines, circDPP4 expression was found to be up-regulated in PC tumors and to predict a lower overall patient survival rate [178]. Reduction of circDPP4 inhibited PCa cell proliferation and metastasis, while promoting apoptosis rate. Overexpression of miR-564 and inhibition of ZIC2 inhibited PCa progression. CircDPP4 functions as a miR-564 sponge and regulates the expression of the miR-564 target gene, ZIC2. Tumor growth is inhibited by silencing of circDPP4, which is accompanied by elevated miR-564 and attenuation of Ki-67 and ZIC2.

#### Drug resistance

Androgen deprivation therapy (ADT) induced by drugs such as enzalutamide (Enz), can lead to longer survival in castration-resistant prostate cancer (CRPC). However, most patients develop drug resistance with prolonged dosing. Increased expression of the AR splice variant ARv7 may play a key role in the development of Enz resistance in CRPC. CircRNA17 expression is lower in PCa patients with higher Gleason scores. In vitro cell line studies demonstrated CRPC C4–2 anti-Enz cells (EnzR-C4–2) to express less of the molecule than parental Enz-sensitive (EnzS-C4-2) cells [179]. Inhibition of circRNA17 in EnzS-C4–2 cells increased ARv7 expression, which lead to increased Enz resistance and cell invasiveness. Mechanistic analysis showed that Enz suppressed the expression of circRNA17 at the transcriptional level by inhibition of host gene, PDLIM5, transcription. CircRNA17 also regulates the expression of ARv7 by altering the expression of miR-181c-5p, which involves miR-181c-5p and ARv7 direct binding to the 3’UTR. Circ0001275 levels are highly up-regulated in enzalutamide-resistant cell lines, with overexpression shown to increase resistance to enzalutamide [180]. CircXIAP expression was upregulated in DTX-resistant PCa tissue specimens and cell lines [181]. CircXIAP is also overexpressed in exosomes of DTX-resistant cells. CircXIAP knockout enhanced DTX sensitivity by inhibiting the proliferation, migration, and invasion of DTX-resistant cells, as well as inducing cell cycle arrest and apoptosis. CircXIAP directly targets miR-1182, down-regulating miR-1182 in DTX-resistant cells and reversing the effects of circXIAP knockout. TPD52 is a target of miR-1182, with up-regulation attenuating the effect of miR-1182 on DTX sensitivity. Importantly, circXIAP deletion suppresses tumor growth and increases DTX sensitivity in vivo.

**Table 4:** Overview of deregulated circRNAs in prostate cancer

CircRNA	Expression	Function	Target microRNA	miRNA target genes/protein	Ref
CircPRKCI	Up	Proliferation(+) Metastasis(+)	miR-24-3p	-	[165]
Circ102004	Up	Proliferation(+) Metastasis(+) apoptosis(−)	-	-	[164]
Circ0003258	Up	Metastasis(+)	miR-653-5p	ARHGAP5	[166]
Circ0004296	Down	Proliferation(-) Metastasis(-)	-	EIF4A3/ETS1	[167]
Circ17	Down	Resistance(-)	miRNA-181c-5p	ARv7	[179]
CircTRPS1	Up	Proliferation(+) Metastasis(+)	miR-124-3p	EZH2	[157]
Circ0074032	Up	Proliferation(+) Metastasis(+) Apoptosis(−)	miR-198	HOXA1	[172]
Circ0062019	Up	Proliferation(+) Metastasis(+) Apoptosis(−)	miR-1253	NRBP1	[173]
CircPAPPA	Up	Proliferation(+) Metastasis(+) Apoptosis(−)	miR-515-5p	FKBP1A	[158]
Circ0076305	Up	Metastasis(+) Apoptosis(−)	miR-411-5p	PGK1	[159]
CircSLC19A1	Up	Metastasis(+) Apoptosis(−)	miR-497	SEPT2	[160]
Circ0062020	Down	Metastasis(-) Resistance(-)	miR-615-5p	TRIP13	[168]
CircXIAP	Up	Proliferation(+) Metastasis(+) Apoptosis(−) Resistance(-)	miR-1182	TPD52	[181]
Circ0016068	Up	Metastasis(+)	miR-330-3p	BMI-1	[169]
Circ0088233	Up	Proliferation(+) Metastasis(+) Apoptosis(−)	miR-185-3p	E2F1/WNT2B	[174]
Circ0057558	Up	Proliferation(+)	miR-206	USP33/c-Myc	[161]
Circ0062019	Up	Proliferation(+) Metastasis(+)	miR-195-5p	HMGA2	[162]
Circ0075542	Down	Proliferation(-) Metastasis(-)	miR-1197	HOXC11	[175]
Circ0030586	Up	Proliferation(+) Metastasis(+)	miR-145-3p	-	[170]
CircFMN2	Up	Proliferation(+) Metastasis(+)	miR-1238	LHX2	[163]
Circ0057553	Up	Proliferation(+) Metastasis(+) Apoptosis(−)	miR-515-5p	YES1	[176]
Circ0006404	Up	Proliferation(+) Metastasis(+) Apoptosis(−)	miR-1299	CFL2	[177]
CircDPP4	Up	Proliferation(+) Metastasis(+) Apoptosis(−)	miR-564	ZIC2	[178]
Circ0001686	Up	Proliferation(+) Metastasis(+)	miR-411-5p	SMAD3 /TGFBR2	[171]
Circ0001275	Up	Resistance(+)	-	-	[180]

## CircRNAs are potential diagnostic and prognostic biomarkers of urinary system tumors

### CircRNAs are potential diagnostic and prognostic biomarkers for kidney Cancer

Aberrant expression of circRNAs is always associated with poor prognosis in urinary system cancer (Table 5). CircPPP6R3 is highly expressed in clear cell renal cell carcinoma tissues and cell lines and may serve as a diagnostic marker for ccRCC [182]. High expression levels of circPPP6R3 are positively related to higher histological grade, T stage, M stage, and advanced clinical stage in ccRCC patients. Furthermore, KM analysis showed that patients with RCC and low levels of circCYP24A1 had shorter overall survival, higher tumor burden, and higher tumor grade [183]. Deficiency of circCYP24A1 in RCC is associated with worse clinical outcomes. High expression levels of circ0085576 are significantly associated with tumor size, clinical stage, metastatic status, and poor survival [184]. Circ0085576 is found to be upregulated in ccRCC tissues and cell lines, thus circ0085576 may serve as a predictor of clinical prognosis in ccRCC patients. A study showed that circ0065217 is abundantly expressed in RCC tissues and cell lines, and its expression is related to advanced TNM stage, large tumor size, and lymph node metastasis [185]. CircAMOTL1L expression is down-regulated in RCC tissues and cell lines [186]. Decreased expression of circAMOTL1L in RCC patients is associated with tumor stage, metastasis, and poor prognosis. Another study found that increased expression of circPRRC2A is positively associated with advanced clinical stage and poor survival in RCC patients [187]. High levels of circPTCH1 were significantly associated with worse patient survival, higher Fuhrman grade, and higher risk of metastasis [188].

#### CircRNAs are potential diagnostic and prognostic biomarkers of bladder Cancer

Dysregulated circRNA expression is closely related to the clinic-pathological features of BCa patients (Table 5). Accurate BCa prognosis for patients guides treatment decisions, thereby improving outcomes for patients.

CircSMARCA5 is up-regulated in BCa tissues compared to adjacent tissues and is associated with larger tumor size, higher tumor stage, and lymph node metastasis [189]. High expression of circSMARCA5 is associated with shorter disease-free survival (DFS) and overall survival (OS), and may serve as a potential prognostic marker for BCa patients. Sequencing of bladder cancer samples found that circ0077837 and circ0004826 are significantly downregulated and significantly associated with worse clinico-pathological features and a poor prognosis in BCa patients [190]. Furthermore, circ0139402 and circ0014130 are highly expressed in bladder cancer samples and correlated with aggressive features and poor survival [191, 192]. A study found that circ0001361 is expressed at high levels in BCa tissues and cell lines, with a positive relationship found for pathology grade and muscle infiltration [193]. Kaplan-Meier survival analysis showed that BCa patients with high levels of circ0001361 expression had lower overall survival rates. Screening and validation in 30 normal and 116 BCa samples revealed circ0137439 to be significantly up-regulated in BCa samples [194]. ROC curves are used to assess the diagnostic value of circ0137439. The Kaplan-Meier method is used to evaluate the prognostic significance of circ0137439 in BCa. Increased expression of circ0137439 is associated with higher tumor stage, higher tumor grade, higher lymph node status, and history of muscle-invasive bladder cancer (MIBC). Circ0137439 can be used to not only differentiate BCa from control, but also to differentiate MIBC from non-muscle invasive bladder cancer. Circ0137439 urine levels can be used as an independent prognostic predictor of recurrence-free survival and overall survival of BCa patients.

#### CircRNAs are potential diagnostic and prognostic biomarkers of prostate Cancer

Screening and early diagnosis of PCa are critical to improved treatment outcomes and reducing patient morbidity (Table 5). Each has been identified as a research priority. Circ0086722 is highly expressed in PCa tissues and cells, with highly levels of circ0086722 is positively correlated with pT3 stage, higher sample Gleason score (> 7) and worse biochemical recurrence-free survival in PCa patients [195]. CircPDHX is highly expressed in PCa tissues and cells, with increased expression of circPDHX related to Gleason score and pathogenic T-stage and serves as an independent prognostic factor for poor survival in PCa patients [196]. Among 56 paired tissues, circSOBP expression is lower in PCa tissues compared to paraneoplastic tissues [197]. The ROC curve showed that the U1 small nuclear ribonucleoproteins of circSOBP was 0.763, indicating that circSOBP is a promising biomarker for differentiating PCa tissues. Low expression of circITCH in tumor tissue is associated with a high risk of advanced pathological T-stage and lymph node metastasis in patients with prostate cancer [198]. CircITCH is able to effectively distinguish tumor tissue from paired adjacent tissue with an Area under the curve（AUC） of 0.812 (95% CI: 0.780–0.845). Patients with high circITCH expression had longer DFS and OS, suggesting that it is a good diagnostic and prognostic biomarker.

**Table 5:** CircRNAs are Potential Diagnostic and Prognostic Biomarkers in Urinary System Tumors

CircRNAs	Tumor Type	Expression in Tumor	Clinical relevance	Ref
CircPPP6R3	RCC	Upregulated	Diagnostic	[182]
CircCYP24A1	RCC	Downregulated	Prognosis	[183]
Circ0085576	RCC	Upregulated	Prognosis	[184]
Circ0065217	RCC	Upregulated	Prognosis	[185]
CircAMOTL1L	RCC	Downregulated	Prognosis	[186]
CircPRRC2A	RCC	Upregulated	Prognosis	[187]
CircPTCH1	RCC	Upregulated	Prognosis	[188]
CircSMARCA5	BCa	Upregulated	Prognosis	[189]
Circ0077837	BCa	Downregulated	Prognosis	[190]
Circ0004826	BCa	Downregulated	Prognosis	[190]
Circ0139402	BCa	Upregulated	Prognosis	[191]
Circ0014130	BCa	Upregulated	Prognosis	[192]
Circ0001361	BCa	Upregulated	Prognosis	[193]
Circ0137439	BCa	Upregulated	Diagnostic/Prognosis	[194]
Circ0086722	PCa	Upregulated	Prognosis	[195]
CircPDHX	PCa	Upregulated	Prognosis	[196]
CircSOBP	PCa	Downregulated	Diagnostic	[197]
CircITCH	PCa	Downregulated	Diagnostic/Prognosis	[198]

### CircRNAs Show Promise as a clinical therapeutic target

As circRNAs have shown an important role in the pathogenesis and progression of urological tumors, there has been an increasing number of studies using circRNAs as clinical therapeutic targets [199]. ciRS-7 is highly expressed in RCC tissues and is associated with high Fuhrman grading and poor survival [71]. PBAE/si-ciRS-7 nanocomplexes are effective for RCC progression and metastasis, therefore targeting ciRS-7 of PBAE/si-ciRS-7 nanocomplexes for drug development may be a promising strategy for RCC gene therapy [71]. CircSNX6 is highly expressed in sunitinib-resistant renal cancer cells [200]. CircSNX6 is found to promote sunitinib resistance in RCC in a cohort sample analysis. CircSNX6 is found to regulate intracellular lysophosphatidic acid levels and promote sunitinib resistance. It is promising to be a new prognostic indicator and a promising therapeutic target. CircME1 interacts with U1 snRNP to upregulate ME1 expression and thereby promote resistance to sunitinib in renal cancer cells [201]. The study suggests that circME1 may be a promising biomarker for predicting sunitinib resistance and a therapeutic target for ccRCC. High circ0008399 expression is associated with poor prognosis in patients with bladder cancer [154]. Targeting the circ0008399/WTAP/TNFAIP3 axis enhances the efficacy of Cisplatin. Regulation of target RNA expression by m^6^A modification reduces the sensitivity of cisplatin in bladder cancer and has potential therapeutic value in bladder cancer. Circ0001361 is expressed at high levels in BCa tissues and cell lines and exerts oncogenic effects in BCa invasion and metastasis by targeting the miR-491-5p/MMP9 axis and is expected to be a potential new target for BCa therapy [193]. HnRNP-L can regulate circCSPP1 to induce autophagy in tumor cells, leading to prostate tumor progression. CircCSPP1 has future promise for the clinical prognosis of PCa and for the development of new therapeutic options [202]. CircFoxo3 inhibits PCa progression and alleviates the sensitivity of PCa cells to doxorubicin by enhancing Foxo3 expression [203]. Targeting circFoxo3/Foxo3/EMT may provide an applicable strategy for exploring potential prostate cancer prognosis and therapeutic approaches.

However, the clinical application of circRNAs in urologic tumors remains largely unexplored, and further research is needed before circRNAs can truly enter clinical practice. On the one hand, a large number of samples, multiple centers, and independent external cohort validation are required for circRNA to become a widely accepted biomarker. On the other hand, the number of cirRNAs reported to affect tumors is still very small, and only a few circRNAs have clarified their biological functions and molecular mechanisms in urological tumors, and more cirRNAs need to be explored.

## Conclusions

This paper reviews the biogenesis and regulation of circRNAs and describes the detection methods and the main functions of circRNAs in vivo. The effects of circRNAs on tumor proliferation, metastasis, apoptosis, metabolism and drug resistance in kidney, bladder, and prostate cancers are summarized. Some potential clinical applications of circRNAs are identified.

In recent years, the development of new detection technologies has facilitated our understanding of previously unknown circRNAs. More and more circRNAs have been found to play an important role in the development and progression of urologic tumors. CircRNA-based tumor diagnosis and treatment options have received much attention, with results suggesting their useful clinical application. However, the current academic understanding of circRNAs is lacking, with a complete appreciation of the importance of circRNA still in its infancy. Our understanding of the role of circRNAs in tumorigenesis and development is not comprehensive. Most current studies of the role and mechanism of action of circRNAs in cancer are limited to single or a few circRNAs, which limits a systematic, comprehensive, and integrated understanding of their biological importance.

Looking ahead, circRNAs require continuous validation and development, if they are to be truly useful for clinical diagnosis and treatment. Current studies do not include clinical data for large cohorts and as such requires further validation. The recent development of single-cell RNA-seq and CRISPR-Cas-based editing technologies will provide for a deeper understanding of circRNAs.

## Electronic supplementary material

Below is the link to the electronic supplementary material.

Supplementary Material 1

## Data Availability

Not applicable.

## Abbreviations

CircRNA: Circular RNA
ccRCC: Clear cell renal cell carcinoma
RCC: Renal cell carcinoma
BCa: Bladder cancer
PCa: Prostate cancer
NcRNA: Non-coding RNA
LncRNA: Long-stranded non-coding RNA
EcircRNA: Exon circRNA
EIcircRNA: Exon-intron circRNA
CiRNA: Circular intronic RNA
TricRNA: tRNA intronic circular RNA
Pre-tRNAs: Precursor transfer RNAs
RBP: RNA-binding proteins
MiRNA: microRNA
RCA: Rolling circle amplification
FISH: Fluorescence in situ hybridization
DSN: Duplex-specific nucleases
AR: Androgen receptor
U1 snRNPs: U1 small nuclear ribonucleoproteins
HCC: Hepatocellular carcinoma
IRES: Internal ribosome entry site
m^6^A: N6-methyladenosine
EMT: Epithelial-mesenchymal transition
Erα: Estrogen receptorα
EVs: Extracellular vesicles
SEPT2: Septin 2
TRIP13: Thyroid hormone receptor interactor 13
BMI-1: B lymphoma Moloney mouse leukemia virus insertion region-1
ADT: Androgen deprivation therapy
Enz: Enzalutamide
CRPC: Castration-resistant prostate cancer
DFS: Disease-free survival
OS: Overall survival
MIBC: Muscle-invasive bladder cancer
AUC: Area under the curve
