# Correction to: The function and mechanisms of action of circular RNAs in Urologic Cancer

**DOI:** 10.1186/s12943-023-01774-2

**Published:** 2023-04-24

**Authors:** Zi-hao Zhang, Yue Wang, Ya Zhang, Sheng-Feng Zheng, Tao Feng, Xi Tian, Mierxiati Abudurexiti, Zhen-Da Wang, Wen-Kai Zhu, Jia-Qi Su, Hai-Liang Zhang, Guo-Hai Shi, Zi-Liang Wang, Da-Long Cao, Ding-Wei Ye

**Affiliations:** 1 Qingdao Institute, School of Life Medicine, Department of Urology, Fudan University Shanghai Cancer Center, Fudan University, Qingdao, 266500 China; 2 Department of Urology, State Key Laboratory of Genetic Engineering, Collaborative Innovation Center for Genetics and Development, School of Life Sciences, Fudan University Shanghai Cancer Center, Fudan University, Shanghai, 200433 China; 3 Department of Oncology, Shanghai Medical College, Fudan University, Shanghai, 20032 P.R. China; 4 Department of Nephrology, Xin Hua Hospital, Shanghai Jiao Tong University School of Medicine, 1665 Kongjiang Road, Shanghai, 200092 China; 5 Shanghai Pudong New Area Gongli Hospital, Shanghai, 200135 China; 6 Institute of Cancer Research, Department of Gynecology, Shanghai Municipal Hospital of Traditional Chinese Medicine, Shanghai University of Traditional Chinese Medicine, Shanghai, 200071 P. R. China

**Correction to: Mol Cancer 22, 61 (2023)**

10.1186/s12943-023-01766-2

Following publication of the original article [1], the authors reported there should have three corresponding authors for this article. The corresponding authors are “Ding-wei YE”, “Da-long Cao”, and “Zi-liang Wang”.

This has been updated above and the original article has been corrected.
