# Supplementary material for: The function and mechanisms of action of circular RNAs in Urologic Cancer
Source: Mol Cancer. 2023 Mar 25;22:61. doi: 10.1186/s12943-023-01766-2 (PMC10039696; doi:10.1186/s12943-023-01766-2)
Supplement: Supplementary file 1 — Supplementary Material 1 [file 12943_2023_1766_MOESM1_ESM.docx]

Figure1. Biogenesis and function of circular RNA (circRNA). Pre-mRNA can undergo either canonical splicing to generate a linear RNA with exon inclusion or back-splicing to produce both a circular RNA and an alternatively spliced linear RNA with exon exclusion. (A) The biogenesis of circRNAs. Biogenesis of circRNAs generates three types of circRNAs: EcircRNAs, ciRNAs, and EIciRNAs. The formation of ecircRNAs is usually by direct back splicing. Direct back splicing is a common shearing method in which precursor-mRNA is looped by linking the 5′ splice donor site downstream of the exon to the 3′ splice acceptor site upstream via covalent bonding. EIcircRNAs also use the method of direct back splicing, but in the process of back splicing, some intron sequences are not removed but remain within the circRNA. The formation of ciRNAs is thought to result from lasso formation from introns removed during pre-mRNA splicing. TricRNA is a circular RNA produced by tRNA precursor splicing. (B) The function of circRNAs. CircRNAs enhance the transcription and splicing of their parental genes by interacting with RNA pol II or U1 small nuclear ribonucleoproteins (U1 snRNPs) . CircRNAs have basic functions as microRNA (miRNA) sponges, RNA-binding proteins (RBP), and templates for regulating transcription and protein translation. CircRNAs can function as microRNA sponges or decoys, protecting target mRNAs from miRNA-dependent degradation. circRNAs containing RBP binding motifs may function as sponges or decoys for these proteins and indirectly regulate their functions. circRNAs have been shown to function as protein scaffolds, facilitating the colocalization of enzymes and their substrates. CircRNA with internal ribosomal entry site elements may be translated under certain circumstances to produce unique peptides.

Figure2. Biological functions of circRNAs in kidney cancer.

(A) CircRNAs are involved in the cell proliferation and cycle of kidney cancer. CircCHT15, circSCARB1, circMTO1, and circEGLN3 affect the cell proliferation of kidney cancer cells by regulating the expression of miRNAs and related proteins. (B) CircRNAs are involved in the metastasis and EMT of kidney cancer. CircPSD3, circRAPGEF5, circUBAP2, and circTLK1 affect the cell metastasis and EMT of kidney cancer cells by regulating the expression of miRNAs and related proteins. (C) CircRNAs are involved in the apoptosis of kidney cancer. Circ0054537, circ001842, circEGLN3, and circNUP98 affect the cell apoptosis of kidney cancer cells by regulating the expression of miRNAs and related proteins. (D) CircRNAs are involved in the cell metabolism of kidney cancer. Circ0035483, circFOXP1, and circ0054537 affect the cell metabolism of kidney cancer cells by regulating the expression of miRNAs and related proteins.

Figure3. Biological functions of circRNAs in bladder cancer (BCa).

(A) CircRNAs are involved in the cell proliferation and cycle of BCa. CircSETD3, circ0068307, circ0030586, and circ0041103 affect the cell proliferation of BCa cells by regulating the expression of miRNAs and related proteins. (B) CircRNAs are involved in the metastasis and EMT of BCa. CircTRPS1, circKDM4C, circITCH, and circACVR2A affect the cell metastasis and EMT of BCa cells by regulating the expression of miRNAs and related proteins. (C) CircRNAs are involved in the apoptosis of BCa. CircCEP128, circ001418, circ0058063, and circ0000326 affect the cell apoptosis of BCa cells by regulating the expression of miRNAs and related proteins. (D) CircRNAs are involved in the drug resistance of BCa. Circ102336, circ0008399, and circ0058063 affect the drug resistance of BCa cells by regulating the expression of miRNAs and related proteins.

Figure4. Biological functions of circRNAs in prostate cancer (PCa).

(A) CircRNAs are involved in the cell proliferation and cycle of PCa. CircTRPS1, circPAPPA, circ0076305, and circSLC19A1 affect the cell proliferation of PCa cells by regulating the expression of miRNAs and related proteins. (B) CircRNAs are involved in the metastasis and EMT of PCa. CircPRKCI, circ0062020, circ0004296, and circ0016068 affect the cell metastasis and EMT of PCa cells by regulating the expression of miRNAs and related proteins. (C) CircRNAs are involved in the apoptosis of PCa. Circ0074032, circ0062019, circ0088233, and circ0075542 affect the cell apoptosis of PCa cells by regulating the expression of miRNAs and related proteins. (D) CircRNAs are involved in the drug resistance of PCa. CircRNA17 and circXIA affect the drug resistance of PCa cells by regulating the expression of miRNAs and related proteins.
